# Comprehensive evaluations of individual discrimination, kinship analysis, genetic relationship exploration and biogeographic origin prediction in Chinese Dongxiang group by a 60-plex DIP panel

**DOI:** 10.1186/s41065-023-00271-2

**Published:** 2023-03-29

**Authors:** Man Chen, Wei Cui, Xiaole Bai, Yating Fang, Hongbin Yao, Xingru Zhang, Fanzhang Lei, Bofeng Zhu

**Affiliations:** 1 Guangzhou Key Laboratory of Forensic Multi-Omics for Precision Identification, School of Forensic Medicine, Southern Medical University, Guangzhou, 510515 China; 2 School of Basic Medical Sciences, Anhui Medical University, Hefei, 230031 Anhui China; 3 Belt and Road Research Center for Forensic Molecular Anthropology, Key Laboratory of Evidence Science of Gansu Province, Gansu University of Political Science and Law, Lanzhou, 730070 China; 4 Key Laboratory of Shaanxi Province for Craniofacial Precision Medicine Research, College of Stomatology, Xi’an Jiaotong University, Xi’an, 710004 China

**Keywords:** Deletion/insertion polymorphism, Individual discrimination, Kinship analysis, Biogeographic origin prediction, Artificial intelligence algorithm

## Abstract

**Background:**

Dongxiang group, as an important minority, resides in Gansu province which is located at the northwest China, forensic detection system with more loci needed to be studied to improve the application efficiency of forensic case investigation in this group.

**Methods:**

A 60-plex system including 57 autosomal deletion/insertion polymorphisms (A-DIPs), 2 Y chromosome DIPs (Y-DIPs) and the sex determination locus (Amelogenin) was explored to evaluate the forensic application efficiencies of individual discrimination, kinship analysis and biogeographic origin prediction in Gansu Dongxiang group based on the 60-plex genotype results of 233 unrelated Dongxiang individuals. The 60-plex genotype results of 4582 unrelated individuals from 33 reference populations in five different continents were also collected to analyze the genetic background of Dongxiang group and its genetic relationships with other continental populations.

**Results:**

The system showed high individual discrimination power, as the cumulative power of discrimination (CPD), cumulative power of exclusion (CPE) for trio and cumulative match probability (CMP) values were 0.99999999999999999999997297, 0.999980 and 2.7029E^− 24^, respectively. The system could distinguish 98.12%, 93.78%, 82.18%, 62.35% and 39.32% of full sibling pairs from unrelated individual pairs, when the likelihood ratio (LR) limits were set as 1, 10, 100, 1000 and 10,000 based on the simulated family samples, respectively. Additionally, Dongxiang group had the close genetic distances with populations in East Asia, especially showed the intimate genetic relationships with Chinese Han populations, which were concluded from the genetic affinities and genetic background analyses of Dongxiang group and 33 reference populations. In terms of the effectiveness of biogeographic origin inference, different artificial intelligent algorithms possessed different efficacies. Among them, the random forest (RF) and extreme gradient boosting (XGBoost) algorithm models could accurately predict the biogeographic origins of 99.7% and 90.59% of three and five continental individuals, respectively.

**Conclusion:**

This 60-plex system had good performance for individual discrimination, kinship analysis and biogeographic origin prediction in Dongxiang group, which could be used as a powerful tool for case investigation.

**Supplementary Information:**

The online version contains supplementary material available at 10.1186/s41065-023-00271-2.

## Introduction

Deletion/insertion polymorphism (DIP) is a fragment length polymorphism genetic marker with short insertion or deletion sequence in the nuclear genome. Compared with the traditionally used mini short tandem repeat (miniSTR), the DIP has some advantages of no amplification slippage peak and convenient detection on capillary electrophoresis platform, which suggests good application potentials in forensic individual identification, kinship testing, biogeographic origin inference, genetic background evaluation and other research fields.

In recent years, some researches have constructed several multiplex DIP systems [1–4], microhaplotype panel with multi-DIPs [5], and compound marker systems with DIPs [6–8] to meet the forensic daily requirements such as individual identification, paternity identification and ancestry information prediction [9]. And validation studies of these panels have also demonstrated their good application efficiencies for degradation sample identifications [1, 2, 7], mixture deconvolutions and so on [5, 6]. The 60-plex panel in this study was designed for forensic individual identification, which included 57A-DIPs, two Y-DIPs and the sex determination locus Amelogenin. All the amplicons designed in the 60-plex panel were less than 230 bp, which might be suitable for degradation biological sample. A series of the validation experiments showed that the 60-plex panel had good application efficiencies because of its high sensitivity (input DNA > = 125 pg), good stability and reproducibility [4]. The relatively high polymorphisms of all the selected A-DIP loci and good performance of this 60-plex panel for individual identification were also previously verified in several populations of China, namely Hunan Han (HNH) [10], Guangdong Han (GDH), Chengdu Han (CDH), Hainan Han (CHH) populations [4]; Hainan Li group (HNL) [11], Yunnan Miao group (YNM) [12] and Dingjie Sherpa group (SP) [13].

Dongxiang ethnic group that we are interested in is an important minority nationality in Chinese Gansu province. Its language belongs to the Mongolian language family of Altai language family. According to the China Statistical Yearbook–2021 released by the National Bureau of Statistics, there are 774,947 Dongxiang individuals, mainly living in the foothills of Chinese Gansu province, China (http://www.stats.gov.cn/tjsj/pcsj/rkpc/7rp/indexch.htm). In previous population genetic studies, researchers evaluated the forensic performances of different panels with 9-STRs [14], 15-STRs [15], and 30-DIP [16] loci in Dongxiang group. Among them, the highest CPD was 0.9999999999999999937 observed in the 15-STRs panel [15]. However, those detection panels with stronger individual discrimination power were lack to be applied to the Chinese Dongxiang group. In addition, the forensic application efficiency of kinship testing still needed to be evaluated when introducing new detection panel. The genetic relationships among Dongxiang group and other reference populations have been previously explored with different kinds of molecular genetic markers, and the Dongxiang group displayed relatively close genetic relationships with Chinese Han, Hui, Salar, Mongolian groups, because the gene flow increases along with the development of society, frequently cultural and economic exchanges [14–18]. To evaluate whether the 60-plex panel could play a good role for the forensic application in Dongxiang group is of great significance for improving the evidence strength of case investigation. Furthermore, genetic structure and background estimations are the useful methods to trace the population evolutional event [13].

In this study, we used the 60-plex panel to genotype 233 unrelated individuals of Dongxiang group, explored the genetic polymorphisms of 57 A-DIPs in the group, and evaluated the individual identification efficiency of this panel by calculating the various forensic parameters and cumulative indexes. Based on the obtained allelic frequency data of 57 A-DIP loci in Dongxiang group, the simulated families were used to evaluate the identification efficiency of the full sibling relationship by the method of LR. What’s more, the genetic similarities and differences among Dongxiang group and other 33 reference populations were fully explored through three quantitative genetic distance indicators i.e. pairwise fixation index (*F*_*ST*_) value, *D*_*A*_ value and the informativeness for assignment (*I*_*n*_). The pairwise *F*_*ST*_ value is a measure of population differentiation, as the greater the differentiation index, the greater the difference. *I*_*n*_ is also an important value to assign the informative of each population, in combination with *D*_*A*_ genetic distance value, they are important parameters to evaluate whether populations are closely related with each other [19]. Additionally, several qualitative genetic relationship analysis methods such as principal component analysis (PCA), multidimensional scaling plot (MDS), neighbor joining (NJ) and maximum likelihood (ML) phylogenetic trees. Finally, we analyzed the ancestral information components of all 4815 individuals from the target Dongxiang group and other reference populations. In order to perform the prediction of individual biogeographic origin, we used four artificial intelligence (AI) algorithms including support vector machine (SVM), RF, decision tree (DT), and XGBoost, which all have good performances in multi-classification problems. Among them, SVM solves the multi-classification problems by combining multiple binary classifiers. DT is a nonlinear discriminant model that supports multi-classification problems. RF is an ensemble learning algorithm, which is composed of multiple decision trees. XGBoost, also known as extreme gradient lifting tree, is an implementation of boosting algorithm, which has a very good effect on classification or regression problems [20]. In this study, the above four AI algorithms were applied to build different biogeographic origin inference models to realize the individual biogeographic origin prediction with unknown intercontinental origin from three continents (Africa, Europe and East Asia) or five continents (Africa, Europe, East Asia, America and South Asia), respectively.

## Materials and methods

### Sample preparation and quantification

A total of 233 blood samples on FTA cards were collected from unrelated healthy Dongxiang individuals living in Chinese Gansu province. All the volunteers were informed of the purpose and significance of the study, and signed written informed consents. To protect the privacy of volunteer, all the samples were anonymized by numbering during the experiments. The idea, technical route and implementation of this study were approved by the ethnic committee of Xi’an Jiaotong University Health Science Center, and the approval number is 2019-1039. QIAamp DNA investigator kit (Qiagen, Hilden, Germany) was used to extract genomic DNA from bloodstain card samples. The extracted genomic DNA was quantified by Qubit™ 4.0 (Invitrogen, Carlsbad, USA) with Qubit™ dsDNA HS and BR Assay kit (Invitrogen).

### Amplification and genotyping with 60-plex panel

All the 60 loci including 57 A-DIPs, 2 Y-DIPs and Amelogenin for sex determination were co-amplified using the AGCU InDel 60 panel (AGCU ScienTech Incorporation, Wuxi, China) on the GeneAmp PCR system 9700 Thermal Cycler (Thermo Fisher Scientific, Waltham, Massachusetts, USA) according to the manufacturer’s instruction. The PCR products were separated on the 3500xL Genetic Analyzer (Thermo Fisher Scientific) and analyzed with the GeneMapper ID-X v1.5 software (Thermo Fisher Scientific) according to the manufacturer’s recommendation. The peak threshold of the allele calling was set as 100 relative fluorescence units (RFUs).

### Statistical analysis

Allele frequencies and forensic parameters including the expected heterozygosity (He), observed heterozygosity (Ho), match probability (MP), polymorphism information content (PIC), power of discrimination (PD), power of exclusion (PE) for trio and typical paternity index (TPI) for 57 A-DIPs were calculated with the online tool STR analysis for forensics (STRAF 1.0.5) [21]. The tests of Hardy-Weinberg equilibrium (HWE) and linkage disequilibrium (LD) of 57 A-DIPs in Dongxiang group were constructed with the Arlequin v3.5 based on the DIP genotypes of 233 unrelated individuals [22]. The CPD, CPE and CMP values were directly calculated to verify the individual discrimination effectiveness of the 57 A-DIPs in this panel according to the previous recommendation [23].

The 57 A-DIPs genotype data of 33 reference populations were obtained from the previous studies, of which 26 were from the 1000 Genomes Phase III [24], and Chinese four Han populations from different regions (HNH, GDH, CDH and CHH) [4, 10], HNL [11], YNM [12] and SP [13]. The detail information including abbreviations, locations, linguistic families, sample sizes, continents and previous references of the Dongxiang group and 33 reference populations were represented in Supplementary Table I. To evaluate forensic efficiency for kinship testing by 57 A-DIPs in the panel in Dongxiang group and 12 reference East Asian populations, we simulated 1000 full sibling pairs and 1000 unrelated individual pairs based on the allele frequencies of 57 A-DIPs in corresponding populations, and then calculated the LRs of hypothesis 1 (random two individuals are full sibling pair) and hypothesis 2 (random two individuals are unrelated individual pair) using the Familias v3 [25]. We set five different LR thresholds as the limits of kinship determination, which were called LR limits, and set as 1, 10, 100, 1000 and 10,000, respectively. The probability density distributions of LRs were plotted with *R* v4.2.1 (https://www.r-project.org).

The analyses of molecular variance (AMOVA) and pairwise *F*_*ST*_ values among the Dongxiang group and 33 reference populations were computed with the Arlequin v3.5. Pairwise *D*_*A*_ genetic distances among 34 populations were conducted with DISPAN program [26]. The genetic relationships among Dongxiang group and other reference populations were also evaluated by PCA with *R* v4.2.1 and MDS using SPSS v19.0 software [27]. The NJ and ML phylogenetic trees were conducted on MEGA v10 [28] and TreeMix v1.1, respectively [29]. To further investigate the genetic differences among Dongxiang group and 33 reference populations, the *I*_*n*_ values of 57 A-DIPs were conducted with the INFOCALC v1.1 [29]. Population structure analyses were performed with the model-based software ADMIXTURE v1.3 [30] and visualized with the online tool *R* package pophelper (http://pophelper.com). The predefined ancestral components were set from two to five. Based on four AI algorithms including SVM, RF, DT and XGBoost, four biogeographic origin inference models were constructed with *R* v4.2.1 to predict biogeographic origins of individuals in three continents (Africa, Europe and East Asia) or five continents (Africa, Europe and East Asia, America and South Asia), respectively.

## Results

### HWE and LD tests of 57 A-DIPs in Dongxiang group

The *p* values of HWE tests for 57 A-DIPs in Dongxiang group and 33 reference populations were shown in Supplementary Table II. The 57 A-DIPs were in accordance with HWE in the Dongxiang group after applying the Bonferroni correction (*p* < 0.0009). Within 13 East Asian and five South Asian populations, these 57 A-DIPs were all in line with HWE. However, it was worth noting that several loci such as rs59841142, rs113011930, rs146875868, rs34076006, rs10590825, rs145191158, rs60867863, rs57981446, rs76158822, rs77635204, rs145010051, rs77206391 and rs538690481 were observed to be significant deviations from HWE in the non-Asian populations we explored, and most of the deviation loci were found in African populations. The results of pairwise LD tests for 57 A-DIPs in Dongxiang group were showed in Supplementary Table III. After Bonferroni correction (*p* < 3.1328 × 10^− 5^), there were no LDs among 57 A-DIPs in Dongxiang group.

### Allele frequencies and forensic parameters of 57 A-DIPs in Dongxiang group

**Fig. 1:**
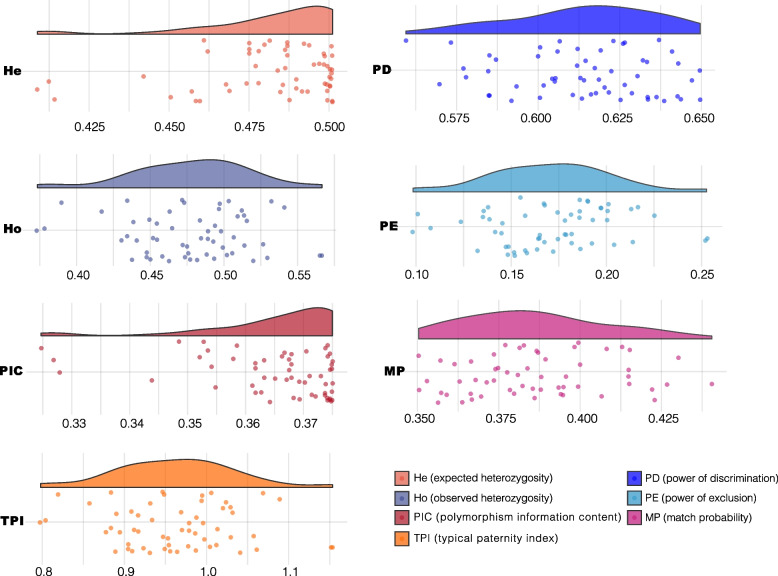
The cloud rain plots of forensic parameters including the expected heterozygosity (He), observed heterozygosity (Ho), match probability (MP), polymorphism information content (PIC), power of discrimination (PD), power of exclusion (PE) for trio and typical paternity index (TPI) of 57 A-DIPs in Dongxiang group

The allelic frequencies of 57 A-DIPs in Dongxiang group were shown in Supplementary Table IV. The average insertion, deletion allelic frequencies of 57 A-DIPs were 0.5045 and 0.4955, respectively. And the insertion allelic frequencies of 57 A-DIPs ranged from 0.3283 (rs1160980) to 0.7146 (rs3834231), whereas the deletion frequencies varied from 0.2854 (rs3834231) to 0.6717 (rs1160980). The forensic parameters (He, Ho, MP, PIC, PD, PE and TPI) of 57 A-DIPs in Dongxiang group were shown in Supplementary Table V, and the raincloud plots were in Fig. 1. The maximum He value was 0.5011 at rs3076465 locus, while the minimum He was 0.4088 at rs3834231 locus; the maximum and minimum of Ho values were 0.5665 (rs5787309), 0.3777 (rs142221201), respectively. The PIC values of 57 A-DIPs ranged from 0.3247 (rs3834231) to 0.3750 (rs3076465), and the average PIC value was 0.3652. The PD, PE, MP and TPI values of 57 A-DIPs varied from 0.5595 (rs3834231) to 0.6496 (rs10590825), 0.1009 (rs142221201) to 0.2526 (rs5787309), 0.3504 (rs10590825) to 0.4405 (rs3834231), and 0.8034 (rs142221201) to 1.1535 (rs5787309), respectively. The CPD, CPE and CMP values of 57 A-DIPs in Dongxiang group were 0.99999999999999999999997297, 0.999980 and 2.7029E^− 24^, respectively. The CPD, CMP and CPE values of 13 East Asian populations were calculated to evaluate the joint application efficiencies of the 57 A-DIPs among East Asian populations and were shown in Table 1. Among the East Asian populations, the CPD values ranged from 0.99999999999999999999997297 in Dongxiang group to 0.999999999999999999998427 in SP group; as for the CPE values, the smallest value was 0.999928 in YNM, whereas the largest value was 0.9999965 in CHS group; the smallest CMP value was observed in Dongxiang group, as the largest value was in SP group (1.5732E^− 22^).

**Table 1:** The cumulative match probability (CMP), cumulative power of exclusion (CPE) and cumulative power of discrimination (CPD) values of 13 East Asia populations based on 57 A-DIPs

Population	CMP	CPE	CPD
**GDX**	**2.7029E**^**− 24**^	**0.999980451**	**0.99999999999999999999997297**
**HNH**	2.90907E^− 24^	0.999974401	0.99999999999999999999997094
**CDH**	3.16174E^−24^	0.999968877	0.99999999999999999999996838
**CHB**	3.58566E^−24^	0.999980563	0.99999999999999999999996414
**GDH**	4.88517E^−24^	0.999981123	0.99999999999999999999995115
**CHH**	4.90264E^−24^	0.99997875	0.99999999999999999999995097
**CDX**	8.27313E^−24^	0.99996374	0.99999999999999999999991727
**JPT**	8.50656E^−24^	0.999978087	0.99999999999999999999991494
**HNL**	1.31852E^−23^	0.999964982	0.9999999999999999999998681
**KHV**	1.85476E^−23^	0.999991051	0.9999999999999999999998146
**YNM**	1.9883E^−23^	0.999927951	0.9999999999999999999998014
**CHS**	2.39649E^−23^	0.999996518	0.9999999999999999999997607
**SP**	1.57317E^−22^	0.999931133	0.999999999999999999998427

Note: *GDX* Gansu Dongxiang, *HNH* Hunan Han, *CDH* Chengdu Han, *CHB* Beijing Han Chinese, *GDH* Guangdong Han, *CHH* Hainan Han, *CDX* Xishuangbanna Dai Chinese, *JPT* Tokyo Japanese, *HNL* Hainan Li, *KHV* Ho Chi Minh Kinh, *YNM* Yunnan Miao, *CHS* Southern Han Chinese, *SP* Sherpa

### Forensic efficiency of the 57 A-DIPs panel for full sibling identification

**Fig. 2:**
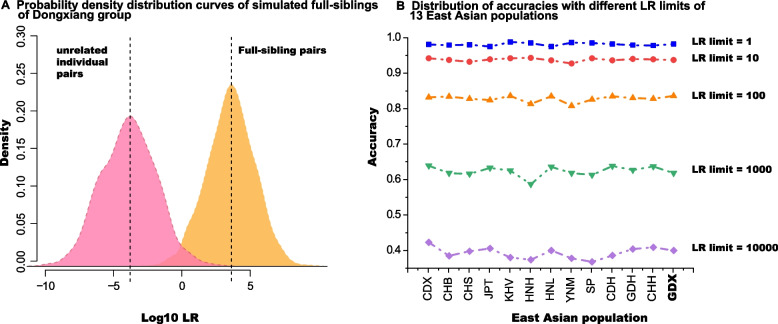
The probability density distribution curves (**A**) of simulated full sibling pairs and unrelated individual pairs of Dongxiang group, and the dotted line plots (**B**) of the accuracy distributions of full sibling identifications with different LR limits (1, 10, 100, 1000 and 10,000) among the Dongxiang group and 12 reference East Asian populations

The LR method was used to evaluate the forensic efficacy of the 57 A-DIPs multiplex panel used in this study for full sibling identification in Dongxiang group and 12 reference East Asian populations. And the probability density curves of simulated full siblings and unrelated individuals of Dongxiang group and the line chats of accuracy distributions with different LR limits (1, 10, 100, 1000 and 10,000) of 13 East Asia populations were shown in Fig.2. The probability density curves of full sibling pairs and unrelated individual pairs showed obvious separation tendency (Fig. 2A). The accuracies and false positive ratios of full sibling identification with different LR limits by 57 A-DIPs panel among 13 East Asia populations were represented in Fig. 2B and Supplementary Table VI, respectively. In Dongxiang group, when the LR limits were set as 1, 10, 100, 1000 and 10,000, the 98.12%, 93.70%, 83.60%, 61.80% and 40.00% of full sibling pairs could be distinguished from the unrelated individual pairs, while the false positive ratios were 2.04%, 0.47%, 0.09%, 0.03% and 0.00%, respectively. The forensic efficiencies of 57 A-DIPs for full sibling identification in other 12 reference East Asian populations were similar to the target Dongxiang group. And the average accuracies of full sibling identifications in 13 East Asian populations were 98.12%, 93.78%, 82,18%, 62.35% and 39.32%, when the LR limits were set as 1, 10, 100, 1000 and 10,000. In a word, the 57 A-DIPs panel showed high accuracies and low false-positive ratios for full sibling identification among East Asian populations.

### Genetic similarities and differences among Dongxiang group and 33 reference populations based on the 57 A-DIPs

To evaluate the role of geographical factors in the formation of genetic differentiations among different populations, we performed AMOVA analysis based on population allelic frequency data. The 12.41% among-group variance could be observed when the 34 populations were divided into five different geographical populations (African, European, South Asian, East Asian and American). And 1.55% variance among populations within continents was found. Furthermore, among 13 East Asia populations, only 2.08% variance among populations and 0.03% variance among individuals within populations could be observed. In the following sections, the PCA, MDS and phylogenetic analyses were also performed to further investigate the genetic relationships between Dongxiang group and 33 reference populations.

### PCA and MDS analyses

**Fig. 3:**
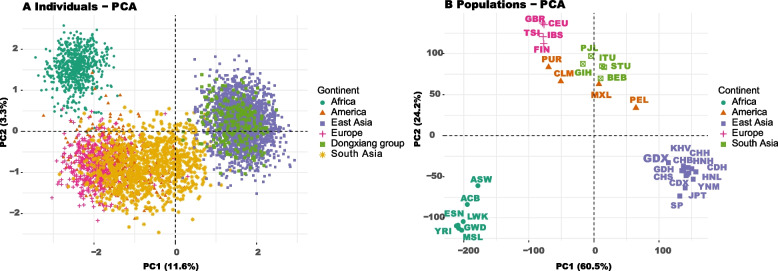
The individual-level PCA (**A**) based on 57 A-DIPs genotypes, and population-level PCA (**B**) based on allele frequencies of 57 A-DIPs in 34 populations

The PCAs of individual-level and population-level were performed based on genotypes, and allelic frequencies of 57 A-DIPs, respectively. And the results mentioned-above were shown in Fig. 3A and 3B, respectively. In PCA at the individual-level, the first two components could explain 14.93% of total variance, of which PC1 accounted for 11.62% and PC2 accounted for 3.31%. The 4815 individuals mainly divided into three clusters, including African individual cluster (malachite green dot), East Asian individual cluster (purple square) and European individual cluster (red cross). The South Asian individuals (yellow star) and American individuals (red triangle) scattered among three main clusters. All Dongxiang individuals belonged to the East Asian cluster in the PCA plot. In population-level PCA based on the allele frequencies, the first two components accounted for 84.70% of the total variance, and the PC1 and PC2 explained 60.50% and 24.20% of the total variance, respectively. In the dimension of PC1, African, European and East Asian populations could be well distinguished, but four American populations and four South Asian populations were closer to those in Asia and Africa, in which Dongxiang group also clustered with East Asian populations. The MDS plots were conducted based on the pairwise *F*_*ST*_ values and *D*_*A*_ genetic distances among Dongxiang group and 33 reference populations, and displayed in Supplemental Fig. 1A and Supplemental Fig. 1B, respectively. In the MDS plots on basis of *F*_*ST*_ values and *D*_*A*_ genetic distances, the distribution patterns of the Dongxiang group and 33 reference populations were consistent with the results of PCA analyses.

The *D*_*A*_ genetic distances and pairwise *F*_*ST*_ values were shown in Supplementary Table VII and Supplementary VIII, respectively. The *D*_*A*_ genetic distances between Dongxiang group and 33 reference populations ranged from 0.0011 (CHB) to 0.0886 (Yoruba in Ibadan, Nigeria, YRI). The average *D*_*A*_ values between Dongxiang group and African, European, American, South Asian, East Asian populations were 0.0780, 0.0379, 0.0235, 0.0178 and 0.0030, respectively. Among the Dongxiang group and 33 reference populations, the remotest genetic relationship with the Dongxiang group was YRI with the biggest pairwise *F*_*ST*_ value (0.1917), whereas the closest genetic relationship was Beijing Han Chinese with the smallest *F*_*ST*_ value (0.0010). The average *F*_*ST*_ value was 0.0088 between Dongxiang group and other 12 East Asian populations. Between Dongxiang group and African, European, American and South Asian populations, the average pairwise *F*_*ST*_ values gradually decreased, which were 0.1772, 0.1116, 0.0694 and 0.0572, respectively.

### NJ and ML phylogenetic trees

**Fig. 4:**
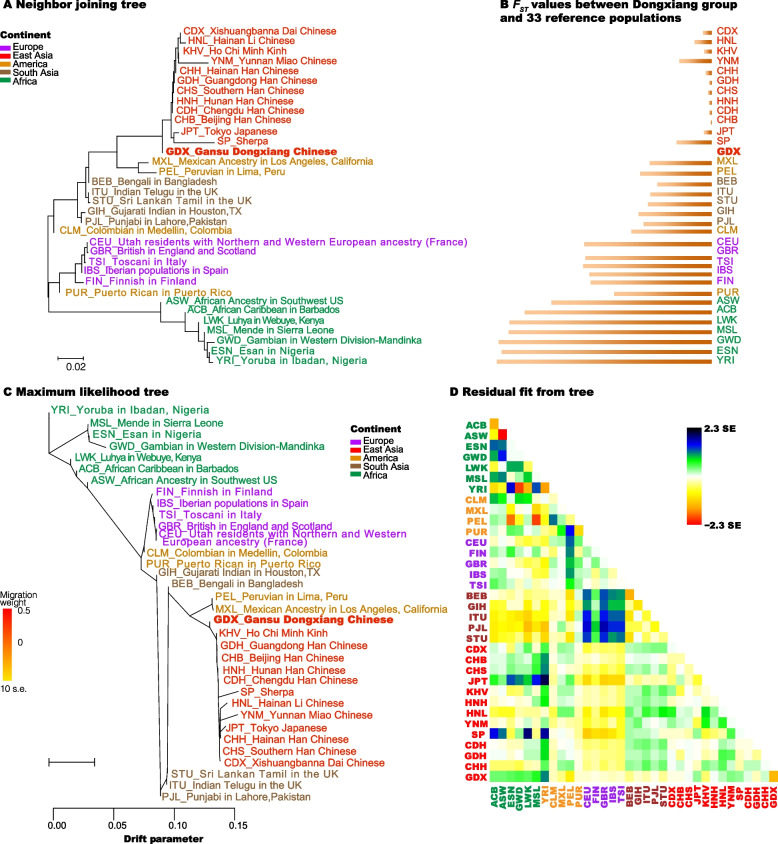
The neighbor joining (NJ) phylogenetic tree (**A**) based on pairwise *F*_*ST*_ values of 34 populations; the horizontal histogram (**B**) of pairwise *F*_*ST*_ values between the target Dongxiang group and 33 reference populations; the maximum likelihood (ML) phylogenetic tree (**C**) based on allele frequencies of 57 A-DIPs in 34 populations; the heatmap (**D**) of residual fit of the ML tree

The NJ phylogenetic tree and histogram of pairwise *F*_*ST*_ values between Dongxiang group and 33 reference populations were shown in Fig. 4A and B, respectively. In the NJ phylogenetic tree, the African, European, South Asian and East Asian populations clustered together and separated from other continental populations, except for the American populations due to their mixed origins. In the branch of 13 East Asian populations, Chinese Han populations of six different regions gathered together; The HNL, Xishuangbanna Dai Chinese (CDX) and YNM groups from Chinese southwest region also clustered into one branch with the Ho Chi Minh Kinh from Vietnam (KHV), SP and Tokyo Japanese (JPT), which clustered into another branch. With the Dongxiang group as the center, most *F*_*ST*_ values also showed an increasing trend with the increase of clade distances, which might indicate that the genetic distances between Dongxiang group and the corresponding populations might be larger, and the phylogenetic relationship might be farther. The ML phylogenetic tree and residual fit from the tree were shown in Fig. 4C and 4D, respectively. The YRI which displayed the largest pairwise *F*_*ST*_ and *D*_*A*_ values with the Dongxiang group was set as the root of the ML tree. The results of ML phylogenetic tree showed that no matter the genetic relationships between Dongxiang group and reference populations, or among different continental populations were similar with the NJ results.

### The *I*_*n*_ values between Dongxiang group and 33 reference populations

The *I*_*n*_ values of 57 A-DIPs among the Dongxiang group and five continental populations were shown in Supplementary Table IX. The maximum average *I*_*n*_ value of 57 A-DIPs between African populations and Dongxiang group was 0.2080 of rs145010051 locus, as the minimum value was 0.001 of rs60564093 locus. And there were 18 loci which showed the average *I*_*n*_ values larger than 0.1, namely rs34287950, rs3067397, rs66739142, rs71852971, rs146875868, rs538690481, rs79225518, rs76158822, rs59841142, rs113011930, rs145191158, rs57981446, rs72085595, rs34076006, rs77206391, rs60867863, rs77635204, and rs145010051. The average *I*_*n*_ values of 57 A-DIPs varied from 0.0007 to 0.0155 in Dongxiang group and the reference populations in East Asia. The average *I*_*n*_ values of 57 A-DIPs in European populations and Dongxiang group ranged from 0.0008 (rs67487831) to 0.2037 (rs145010051). And four loci (rs34287950, rs60867863, rs113011930, rs145010051) were the average *I*_*n*_ values larger than 0.1 in European populations and Dongxiang group. Locus rs145010051 was the only one with the average *I*_*n*_ values larger than 0.1 between Dongxiang group and American, South Asian populations.

### Genetic structure analyses among Dongxiang group and 33 reference populations

#### ADMIXTURE analysis

**Fig. 5:**
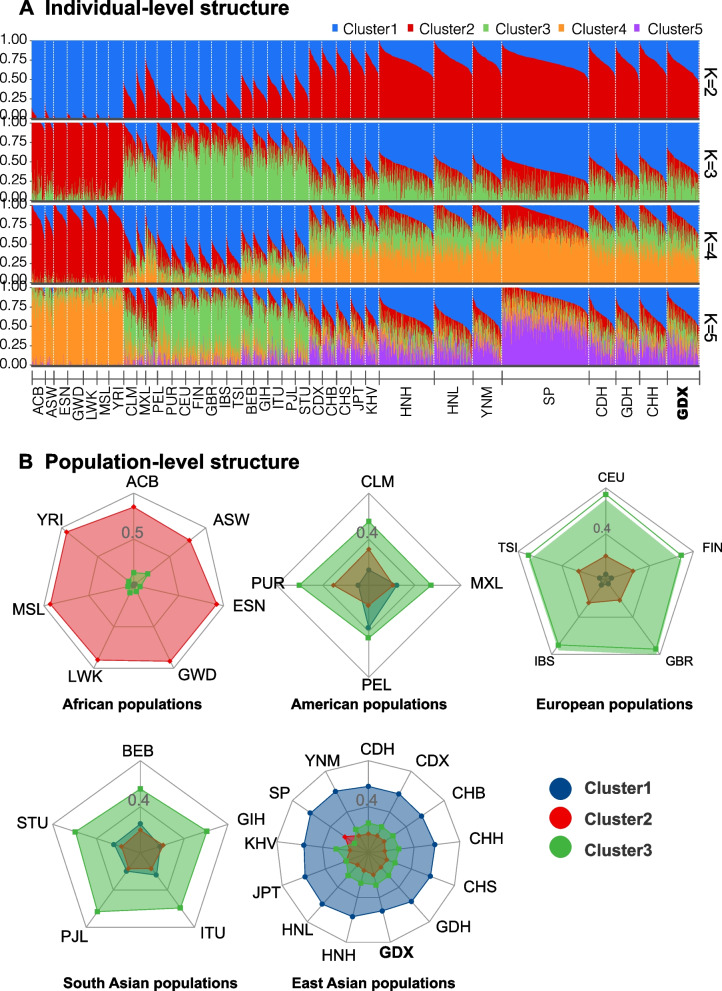
The results of individual-level structure (**A**) and population-level structure (**B**) by the model-based ADMIXTURE analyses

Model-based admixture analysis was performed to further investigate the ancestral information component of the Dongxiang group. The assumed numbers of ancestral components (*K*) were set from two to five, and the genetic structure result of individual-level was shown in Fig. 5A. The radar chart of population-level structure was shown in Fig. 5B, where the ancestor component was set to the optimal value of three. As the *K* was two, there were two ancestral components (the blue and red components), which were roughly derived from the African and non-African populations. When *K* was three, the third ancestral component (the green one) appeared in the American, South Asian and European populations. When *K* were four or five, no other significant ancestral components appeared in five continental populations. However, the proportions of ancestral information components among the American, South Asian and European populations were still different with each other. Among 13 East Asian populations, the genetic structure of SP group was different from other 12 populations, while the Dongxiang group shared the similar pattern with other East Asian populations.

### Biogeographic origin inference based on AI algorithm models

Due to the existence of large differences in allelic frequency distributions among different continents, the 60-plex system possessed the potential to predict individual biogeographical origin. In this study, four AI algorithms (RF, XGBoost, SVM and DT) were used to construct the biogeographic origin inference models based on the genotype data of 57 A-DIPs of 4815 unrelated individuals of Dongxiang group and 33 reference populations. The confusion matrixes and associated statistics of the biogeographic origin inference models for three continental (African, European and East Asian), and five continental populations (African, European, East Asian, American and South Asian) were shown in Table 2 and Supplementary Table X, respectively. When we focused on three continents (Africa, Europe and East Asia), the accuracies and 95% confidence intervals (CI) of biogeographic origin inference of RF, XGBoost, SVM and DT models were 0.997 (95% CI: 0.9912~ 0.9994), 0.993 (95% CI:0.9855 ~ 0.9972), 0.9889 (95% CI:0.9803 ~ 0.9945) and 0.9476 (95% CI: 0.9319 ~ 0.9606), respectively. While all individuals were considered and divided into five continents, the corresponding accuracies of the four prediction models decreased. The accuracies and 95% CI values of biogeographic origin inference of RF, XGBoost, SVM and DT models were 0.8993 (95% CI: 0.8808~ 0.9157), 0.9059 (95% CI: 0.888~ 0.9218), 0.8993 (95% CI: 0.8808 ~ 0.9157), and 0.8135 (95% CI: 0.7903 ~ 0.8351) in five continents, respectively.

**Table 2:** The confusion matrixes and statistics of the RF, XGBoost, SVM and DT models for the three continental (African, European and East Asian) biogeographic origin inferences, respectively

**Random forest (RF)**				**Extreme gradient boosting (XGBoost)**			
Prediction of biogeographic origin	Africa	Europe	East_Asia	Prediction of biogeographic origin	Africa	Europe	East_Asia
Africa	165	2	0	Africa	162	1	0
Europe	0	122	0	Europe	3	123	2
East_Asia	0	1	703	East_Asia	0	1	701
Accuracy of biogeographic origin inference: 0.9970, 95% CI: (0.9912 ~ 0.9994)				Accuracy of biogeographic origin inference: 0.9930, 95% CI: (0.9855 ~ 0.9972)			
**Support vector machine (SVM)**				**Decision tree (DT)**			
Prediction of biogeographic origin	Africa	Europe	East_Asia	Prediction of biogeographic origin	Africa	Europe	East_Asia
Africa	163	3	1	Africa	148	10	4
Europe	1	120	3	Europe	14	103	9
East_Asia	1	2	699	East_Asia	3	12	690
Accuracy of biogeographic origin inference: 0.9889, 95% CI: (0.9803 ~ 0.9945)				Accuracy of biogeographic origin inference: 0.9476, 95% CI: (0.9319 ~ 0.9606)			

## Discussion

In this study, a multiplex panel including 57 A-DIPs, 2 Y-DIPs and Amelogenin was used to genotype 233 unrelated healthy individuals of Dongxiang ethnic group in Chinese Gansu province. We fully assessed the forensic parameters and genetic polymorphisms of 57 A-DIPs; and estimated the forensic efficiencies of individual discrimination and full sibling identification of the 60-plex panel in Chinese Dongxiang group. In addition, the 57 A-DIPs genotype data of 4815 individuals from 33 reference populations were collected to explore the genetic differentiations and relationships between Dongxiang group and other continental populations through the pairwise *F*_*ST*_, *D*_*A*_ genetic distances and *I*_*n*_ value assessments, NJ and ML phylogenetic tree constructions, PCA, MDS and structure analyses, respectively. Finally, based on the genotypes of 57 A-DIPs, four AI algorithms were constructed to predict the biogeographical origins of unknown individuals.

All 57 A-DIP loci were confirmed to meet HWE in Dongxiang group. And there were no LDs among pairwise DIP loci in the group, which indicated that this system had good applicability in the target group. Additionally, several loci (rs59841142, rs113011930, rs146875868, rs34076006, rs10590825, rs145191158, rs60867863, rs57981446, rs76158822, rs77635204, rs145010051, rs77206391 and rs538690481) in this system did not conform to HWE in non-Asian populations (especially in African populations), which also suggested that the system should conduct sufficient population genetic analyses before putting into forensic applications, so as to ensure the reliability of interpretation of subsequent evidence result.

The insertion and deletion alleles of 57 A-DIPs were widely distributed in the Dongxiang group, and all loci were relatively high polymorphisms. Compared with the reference populations in East Asia, the 57A-DIPs system had the lowest CPE (2.7029E^-24^) in Dongxiang group, which indicated that the system had high individual identification effectiveness in the group. The individual identification efficiency of 57 A-DIPs (CPD: 0.99999999999999999999997297) was higher than that of 30 A-DIPs (CPD: 0.999999999985) [16], 9 A-STRs (CPD: 0.999999989) [14] and 15 A-STRs (CPD: 0.9999999999999999937) systems in Dongxiang group [15]. Whether in the target Dongxiang group or in 12 reference East Asian populations, the 57 A-DIPs system showed the good performance of full sibling identification. Although there was a certain probability of false positive result, on average, 98.12%, 93.78%, 82.81%, 62.35% and 39.32% of true full sibling pairs could be identified from unrelated individual pairs when the LRs of hypothesis 1 (full sibling pair) were 1, 10, 100, 1000 and 10,000 times of those ratios of hypothesis 2 (unrelated individual pair). In addition, due to the presence of more polymorphic loci, the identification accuracy of 57A-DIPs system for the full sibling was higher than that of 43A-DIPs system, meanwhile the false-positive rate was also lower [31].

The genetic similarities and differences among Dongxiang group and 33 reference populations were explored by the AMOVA, PCA, MDS analyses, pairwise *F*_*ST*_ values, *D*_*A*_ genetic distances, *I*_*n*_ values assessments, and NJ and ML phylogenetic tree constructions. From the AMOVA analysis, 12.41% of the A-DIP loci diversities could be attributed to geographical aspect. The *F*_*ST*_ and *D*_*A*_ values between Dongxiang group and African, European, American, South Asian, East Asian populations gradually decreased, indicating that Dongxiang group had relatively remote genetic distances with African and European populations, while which had relatively closer genetic distances with East Asian populations. And it could also be verified by the presence of more loci with *I*_*n*_ values larger than 0.1, which indicated the large difference between the two populations [32]. In the NJ and ML phylogenetic trees, the African, European and East Asian populations could well cluster into large branch, whereas the American and South Asian populations with mixed ancestral origins scattered among the East Asian and European branches. The target Dongxiang group clustered with other East Asian populations, showing their closer genetic relationships. The PCA analyses based on individual-level and population-level, the MDS analyses with *F*_*ST*_ and *D*_*A*_ genetic distances also confirmed the close genetic relationships between Dongxiang group and other East Asian populations. Previous researches based on various genetic markers also indicated that Dongxiang group had closer genetic distances with Chinese other groups, such as the Xibo and Salar groups [16], Tibetan ethnic group [16, 33], Hui, Bonan and Yugur groups [34].

From the results of individual-level and population-level ancestral component evaluations by the model-based admixture analyses, the Dongxiang group and other continental reference populations could be significantly divided into three different ancestral components. When the hypothetical ancestral components (*K* values) increased from two to five, the East Asian, American and South Asian populations also showed the slight difference of ancestral components with other continental populations. Due to the genetic differences among different populations, 60-plex system had the potential to predict the unknown individuals' biogeographic origins. Based on the 57 A-DIPs genotypes, when we differentiated three continents (Africa, Europe and East Asia), the highest accuracy of biogeographic origin inference model was observed in RF algorithm, the accuracy of XGBoost algorithm model was the second, and followed by SVM algorithm model and DT algorithm model. While in the more detailed divisions of five continents (Africa, Europe, East Asia, South Asia and America), XGBoost algorithm showed the most accurate prediction result of unknown individuals' biogeographic origins, which was 90.59%. The prediction accuracies of RF algorithm model and SVM algorithm model were the same, and followed by DT algorithm model. In previous study, the XGBoost model performed the best for small ranges of population (Southeast Asian, East Asian and Russian) differentiations through analyzing the input data of genotypes and allelic frequencies of 15 A-DIPs, and genetic distances between populations [35]. The results reminded us that different AI algorithms might have different performances in dealing with different types of input data and classifications with different scales.

## Conclusions

In this study, we genotyped 233 unrelated individuals of Dongxiang group in Chinese Gansu province with a 60-plex system, and verified that the studied 57 A-DIPs had relatively high genetic polymorphisms in Dongxiang group. The obtained results indicated that this system had high forensic application efficiencies in individual identification and full sibling identification in the target Dongxiang group. According to the population genetic analyses among Dongxiang group and 33 reference populations, the Dongxiang group had relatively closer genetic distances with the East Asian populations. Based on the 57 A-DIPs genotypes, the biogeographic origin prediction model of RF algorithm could accurately predict 99.7% individuals' biogeographic origins of three continents (Africa, Europe or East Asia). The XGBoost algorithm model could correctly predict the biogeographic origin information for 90.59% of the five continental individuals (Africa, Europe, East Asia, America and South Asia). In general, this 60-plex system had good performances of individual identification, full sibling identification and biogeographic origin prediction, which could be used as a powerful tool for forensic case investigation.

## Supplementary Information

**Additional file 1: Supplementary Table I.** The detailed information of the target Chinese Gansu Dongxiang group and 33 reference populations. **Supplementary Table II.** The *p* values of Hardy-Weinberg equilibrium (HWE) tests of the 57 A-DIPs among Dongxiang group and 33 reference populations. **Supplementary Table III.** The *p* values of the LD tests of 57 A-DIPs in Chinese Gansu Dongxiang group. **Supplementary Table IV.** The insertion and deletion allelic frequencies of 57 A-DIPs in Chinese Gansu Dongxiang group. **Supplementary Table V.** The forensic parameters (He, Ho, MP, PIC, PD, PE, and TPI) of 57 A-DIPs in Chinese Gansu Dongxiang group. **Supplementary Table VI.** The accuracies and false positive ratios of full sibling identifications with different LR limits (1, 10, 100, 1000 and 10,000) by 57 A-DIPs system among 13 East Asia populations. **Supplementary Table VII.** The pairwise *F*_*ST*_ values between Chinese Gansu Dongxiang group and 33 reference populations. **Supplementary Table VIII.** The pairwise *D*_*A*_ values among Chinese Gansu Dongxiang group and 33 reference populations. **Supplementary Table IX.** The *I*_*n*_ values between Chinese Gansu Dongxiang group and 33 reference populations. **Supplementary Table X.** The confusion matrixes and statistics of the RF, XGBoost, SVM and DT models for the biogeographic origin predictions of the five continents.

**Additional file 2.**

## Data Availability

The raw genotype data used and analyzed during the current study are available from the corresponding author on reasonable request.
