# Supplementary figures and images for: Comprehensive evaluations of individual discrimination, kinship analysis, genetic relationship exploration and biogeographic origin prediction in Chinese Dongxiang group by a 60-plex DIP panel
Source: Hereditas. 2023 Mar 29;160:14. doi: 10.1186/s41065-023-00271-2 (PMC10052841; doi:10.1186/s41065-023-00271-2)

**A  $F_{ST}$  - MDS**

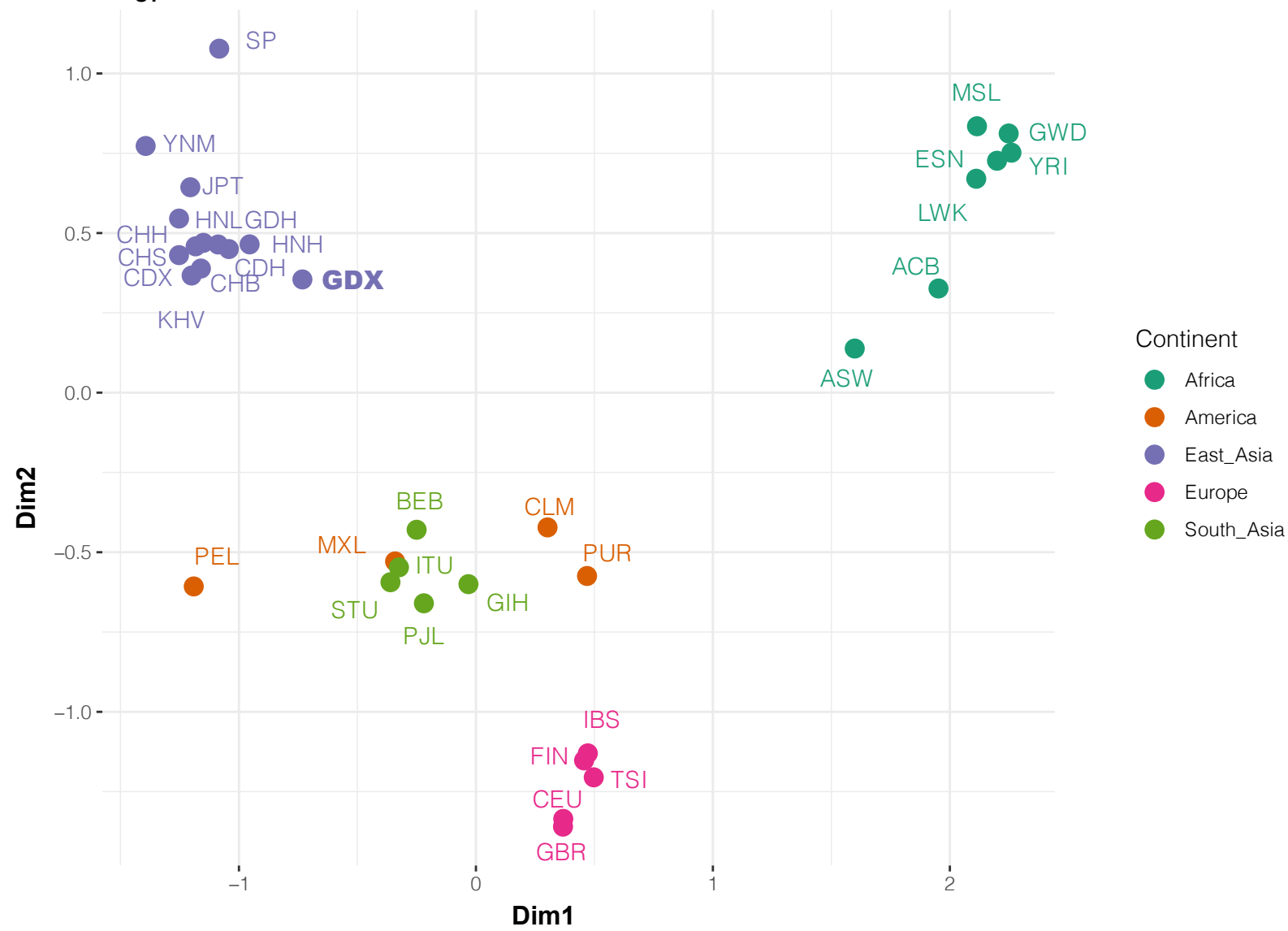

**B  $D_A$  - MDS**

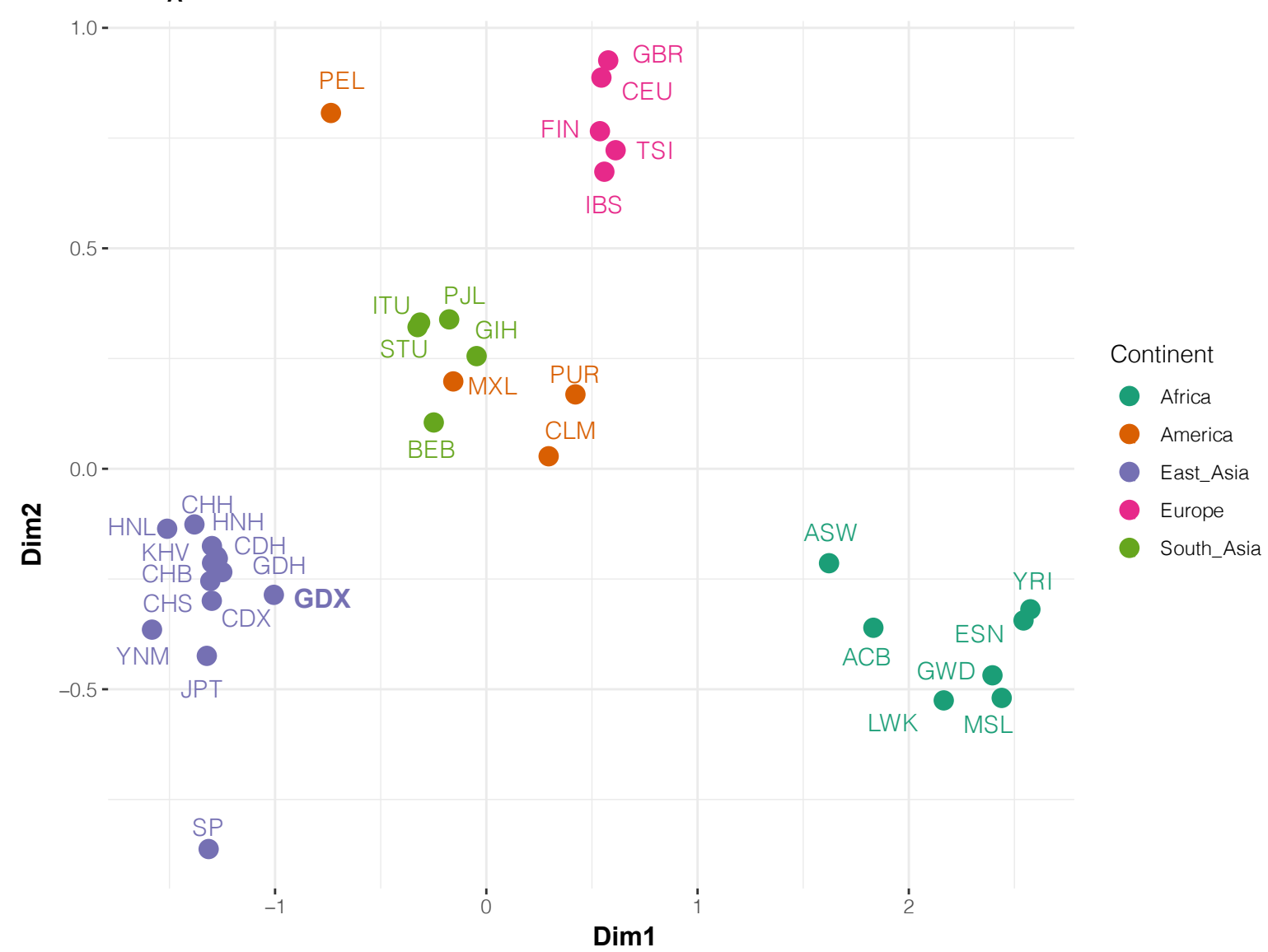

Supplement: Supplementary file 2 — Additional file 2. [file 41065_2023_271_MOESM2_ESM.pdf]
